# Influence of various factors on the legal outcome of cases of child abuse—experiences gathered at an interdisciplinary forensic examination center in Vienna, Austria

**DOI:** 10.1007/s00414-023-03094-y

**Published:** 2023-10-12

**Authors:** Maria Kletečka-Pulker, Klara Doppler, Sabine Völkl-Kernstock, Laura Fischer, Magdalena Eitenberger, Mark Mussner, Sophie Klomfar, Eva Anna Mora-Theuer, Chryssa Grylli, Atanas G. Atanasov, Susanne Greber-Platzer

**Affiliations:** 1 Ludwig Boltzmann Institute for Digital Health and Patient Safety (LBI-DHPS), Ludwig Boltzmann Gesellschaft, Währinger Straße 104/10, 1180 Vienna, Austria; 2 Institute for Ethics and Law in Medicine, University of Vienna, Spitalgasse 2-4, 1090 Vienna, Austria; 3 Department of Children and Adolescent Psychiatry, Medical University Vienna, 1090 Vienna, Austria; 4 Division of Pediatric Pulmonology, Allergology and Endocrinology, Department of Pediatrics and Adolescent Medicine, Medical University Vienna, 1090 Vienna, Austria; 5 Institute of Genetics and Animal Biotechnology of the Polish Academy of Sciences, Polish Academy of Sciences, Jastrzebiec, 05-552 Magdalenka, Poland

**Keywords:** Child maltreatment, Child abuse, Legal proceedings, Forensic examination centers, Court files

## Abstract

**Background and objective:**

To improve the currently low conviction rate in cases of child abuse a forensic examination center for children and adolescents (FOKUS) was established in Vienna, Austria. Besides a state of the art treatment combined with forensic documentation, one of FOKUS’ key goals is to identify potential areas for improvements within the process legal proceedings in cases of child abuse through constant scientific monitoring. The accompanying study at hand includes all patients referred to FOKUS within a two year timeframe (*n* = 233), monitoring their progression from first contact with the medical professionals from FOKUS to the end of criminal proceedings. A detailed analysis of case files was performed in those cases that were reported to the legal authorities by the clinicians of FOKUS (*n* = 87). Aim of the study is to investigate which factors contribute to the initiation of legal proceedings and a successful conviction.

**Results:**

Multivariate logistic regression analyses showed that main proceedings were opened more often in cases where the offender was an adult (*p* < 0.001) or admitted his guilt (*p* < 0.001) and if digital traces were available (*p* = 0.001) or trial support (*p* = 0.024) present. Furthermore, the combined occurrence of medical documentation and victim disclosure was related to a higher probability of opening main trials.

**Conclusion:**

These findings underline how challenging the successful persecution of an offender in cases of child abuse is.

**Supplementary Information:**

The online version contains supplementary material available at 10.1007/s00414-023-03094-y.

## Introduction

On account of the growing awareness in the field of children’s rights, the issue of child abuse and its prevention and detection has become a prominent public health concern. A central focus in this context is the legal prosecution of perpetrators. The goal of thorough legal proceedings is twofold: to protect the victim from further abuse and to hold the perpetrator criminally accountable [1, 2] or, as Walsh phrases it to “move offenders off the street” [3].

### From first suspicion to successful conviction

The multi-step procedure leading up to a conviction of child abuse perpetrators is a notoriously long and complicated process [3, 4] carrying the risk of premature termination at all stages: from the initial step of discovering and reporting a case of suspected child abuse up to a successful conviction [4, 5]. Consequently, “most substantiated and founded child abuse cases do not lead to prosecution” [4].

In order for the judicial process to be set in motion, the abuse of the child must first be discovered and reported to the executive and judicial bodies involved in prosecution. Reporting mechanisms include reports of the victims themselves or reference persons, medical professionals, or child and youth protective services. The process of detecting and reporting child abuse is complicated and fraught with difficulties that originate, among other things, in the high number of abuse cases within the family and an associated high rate of undisclosed cases due to the fact that victims and perpetrators alike may actively conceal psychological and physical injuries [6–8]. Furthermore, in suspected cases, a clear assignment of the injuries to an abuse by medical professionals is a difficult undertaking [9–11]. Merely a small fraction of child abuse cases are reported at all [12, 13].

Medical doctors and nurses play a prominent role in diagnosing and reporting child abuse. This is especially relevant in infancy and early childhood, as pediatricians are often the only professionals who regularly see children of this age group [14]. On account of the important role of medical professionals it is imperative for physicians to continuously keep up to date with recommendations of medical evaluation and the interpretation of findings in cases of suspected child abuse [15]. After all, medical professionals with profound experience and specific certification have increased knowledge and competence in interpreting medical and laboratory findings in children who suffered sexual or physical abuse [16–18].

After a case of child abuse has been reported, it is up to the executive bodies to establish “initial suspicion”. According to the Austrian Code of Criminal Procedure, initial suspicion exists if it can be assumed based on certain indications that a criminal act has been committed. Provided that this prerequisite is met, the case is subjected to a preliminary investigation. Preliminary proceedings serve to clarify the facts of the case and the grounds for suspicion by means of comprehensive and thorough investigations and review of the evidence. If the facts available in the preliminary investigation stage indicate that a conviction in court is likely and there are no grounds for discontinuing the proceedings or withdrawing from prosecution, the case is reviewed and tried before court.

According to a recent Australian study, legal proceedings beyond the preliminary investigation stage are only carried out in 20.8% of cases of suspected child physical abuse and in 16.6% of cases of suspected child sexual abuse (following an initial suspicion of child abuse). It is furthermore assumed that only about 12% of offenses reported to the police result in a conviction [19].

### Evidence in cases of suspected child abuse

Whether a case of suspected child abuse is investigated beyond the investigative stage depends on a number of factors. However, the most significant variable for the initiation of a trial (and the consequent possibility of a conviction of the perpetrator) is the state of evidence.

According to Myers [20], seven different types of evidence can be used to reach conviction in cases of child sexual abuse, including medical evidence, disclosure and trial testimony by child, and evidence that corroborates the abuse such as prior crimes committed by the perpetrator. The principle of “the more, the better” does not seem to apply with these types of evidence used [20]. Blackwell et al. oppose this finding, arguing that there is a noticeable increase in conviction rates if three or more types of evidence can be presented [5]. This seeming contradiction in data could be explained by further examining not only the number of evidence, but the specific types of evidence utilized in legal proceedings. Further studies are necessary to understand this issue [21]. Especially the lack of research in evidence influencing the initiation of main proceedings, rather than focusing solely on the outcome of the trial has to be considered. This is especially relevant due to the incredibly high drop-out rate at this investigation stage (13).

### Status quo and ideas for improvement from an Austrian perspective

The role of medical personnel lies not only in reporting cases of potential child abuse to the authorities, but physicians and nurses also have a vital function in the gathering and preservation of evidence. The importance of medical evidence on the initiation and outcome of legal proceedings has been subject to numerous studies [4, 5, 21–27]. The aim to improve the state of documenting and collecting medical evidence was one of the key motivators for the foundation of FOKUS (forensic examination center for children and adolescents, in German: Forensische Kinder- und Jugenduntersuchungsstelle). FOKUS is set up at the Department of Pediatrics and Adolescent Medicine, Medical University of Vienna at The Vienna General Hospital. During the time of our study, the FOKUS core team consisted of pediatricians, a medico-legal expert, a legal professional as well as psychologists and administrative and scientific personnel. In addition, the forensic examination center worked closely with several other disciplines (pediatric gynecology, pediatric surgery, pediatric, and adolescent psychiatry as well as radiology).

The aim of FOKUS is a timely medical documentation and examination of any injury — including physical examination, additional diagnostic examinations (e.g., radiology, gynecology, ophthalmology), probe sampling, securing of evidence, photographic and descriptive documentation, psychological evaluation, medical/family/social history – that may potentially have their origin in child (sexual or physical) abuse or neglect. The team of FOKUS, together with experts from different professions (pediatricians, medico-legal experts, child psychiatrists and psychologists, child surgeons as well as legal experts), developed a documentary tool for the investigation of different types of abuse, which is meanwhile used in most Austrian hospitals in cases of suspected child abuse. Not only is this method suitable for a better and quicker collection of evidence, thus facilitating the forensic evidence necessary for prosecution, but it also fulfills the potential victim’s need for an immediate and sensitive examination process and thus remedies the usual multitude of examination stages at a later stage.

FOKUS addresses the obstacle that in Austria forensic evaluations are solely assigned to official medico-legal experts, whose work assignment only comprises of collecting and safeguarding evidence for concrete criminal proceedings and thus providing an expert opinion admissible in court. This strict connection of official forensic examination solely with criminal proceedings is problematic, as the forensic experts are often involved at a time where the majority of evidence has already been irrevocably lost [28, 29].

The aim of the study is to examine influences on reporting frequencies and legal proceedings in cases of child abuse on the basis of all cases investigated by FOKUS within the first two years of the examination center’s initiation. A strong focus is placed on the impact of various evidence types in the criminal prosecution process.

## Methods

### Study population

The first two years following the initiation of FOKUS were determined as the evaluation timeframe of the project. In the relevant time period between July 2015 and June 2017, the forensic examination center for children and adolescents dealt with 233 cases of suspected child abuse between the ages of 0 and 18 years.

Of the 233 cases recorded by FOKUS, 87 thereof fulfilled the required premises for reporting and were passed along to the public prosecutors. Consequentially, 73 judicial files of alleged abusive behavior towards children and a total of 87 potential victims could be evaluated in this study. The numeral discrepancy of victims of abuse and judicial files arises from the fact that cases concerning the same alleged perpetrator are summarized under one judicial file, if the multiple committed offences are similar and brought to persecution at the same time.^1^

The numerical discrepancy between the children initially presented to FOKUS (*n* = 233) and the much smaller number of cases that were brought to the judicial bodies through a report by the medical professionals of FOKUS (*n* = 87) can be explained by different underlying reasons: children with most likely accidental injuries (or a severe amount of uncertainty concerning the act of infringement was given) were not reported; furthermore, it is possible that a police report had already been submitted by other parties for example by parents, child, youth welfare officials or school teachers prior to contact with FOKUS. Other factors that have to be taken into account before reporting are a possible guaranteed separation of the perpetrator, the age of the victim, the psychological stability, and well-being of the child.

### Data collection and study team

The study team consisted of experts with a multidisciplinary expertise with backgrounds in social work, law, as well as social and political sciences. At all times, at least one legal professional with profound knowledge of the Austrian judicial system and experience in working with court files was present during the phase of analyzing the physical court files.

Permission for the review of the relevant cases was sought out beforehand by the prosecutor’s office and the court. In Austria, the required case files for penal cases were not yet available in a digitalized form and therefore the analysis of court files had to be carried out directly in the court building.

The study team had access to all files and documents available on each case. Hence, the review included patient frequencies and medical documentation from FOKUS for all cases reported to the police as well as statements from the victims, perpetrators, witnesses, and other involved institutions in context with the abused cases.

A specifically designed checklist of 50 points was used to evaluate the court files that was grouped into 4 categories: (1) specifications on the victim, (2) medical examination, (3) involvement of child protective services or other related bodies, (4) preliminary proceedings and main proceedings.

Each of the categories was divided in several subcategories. In the process of analysis, certain aspects of the checklist proved to be most valuable:

- *Medical Documentation* consisted of a standardized examination sheet, photographic documentation of injuries, specific checklists for different types of abuse, probe sampling, and secure evidence of traces.
- *Digital Traces* consisted of data (messages, photos, diary entries, …) stored on an electronic device and were accepted as evidence by the court.
- *Victim Disclosure* was included if the child’s testimony of abuse was given in front of police or court, but also if included in the statement.
- *Process guidance* was present, when the victim was accompanied by an official organization during criminal proceedings. This service comprises psychosocial and legal support aiming to minimize psychological stress and the risk of re-traumatization during the proceedings.

The types of abuse were first categorized according to the distinctions provided by the FOKUS documentary tool. However, since we encountered exclusively cases of sexual and physical abuse, our analysis focuses specifically on these types of abuse.^2^

Even though the checklist was solely designed for the purpose of data extraction and never intended for publication, all elements that could hint at a person’s identity were carefully anonymized at this early stage of the project.

### Data analysis

The data was analyzed using IBM® SPSS® Statistics, version 26, and R Statistical Software, version 3.5.0.

The independent variables were the sex and age of the victim, as well as the sex and age of the offender, in terms of “adult” or “minor”, type of abuse (sexual abuse or/and physical abuse), victim disclosure, guilty plea, trial support, medical documentation, and digital traces.

The dependent variables were the presence or absence of a police report and the opening or non-opening of a trial in court. Significance level of α = 0.05 was used for inferential statistics.

Following the recommendations of Hosmer Jr et al. [30], the associations between independent and dependent variables were first evaluated individually. In a second step, the significant independent variables were included in a multivariate model.

In the utilized data set, there was a lack of independence between the data sets in some cases, as there were several siblings who had the same offender. To take this into account, the univariate analyses were performed using Pearson’s chi-square test with second-order correction of Rao and Scott [31]. The R package “srvyr” was used for this step. The multivariate analysis was performed with a logistic regression with clustered robust standard errors. For this purpose, the R packages “sandwich” [32, 33], “lmtest” [34] and “DescTools” [35] were used.

In the multivariate analysis concerning the dependent variable “opening of proceedings”, the *Firth-Correction* was used for the logistic regression due to the small samples with rare events. This is a penalized maximum likelihood estimation [36–38], which was performed with the R package “brglm” [39].

Nagelkerke pseudo-R^2^ values were given as measures of goodness of fit for the logistic regressions. It was verified that there were no influential cases in terms of leverage, that the linearity of the logit for continuous predictors was present and that there was no multicollinearity between the predictors of the models [30].

If an association that was significant in a bivariate analysis failed significance in the multivariate analysis, then a mediation analysis was performed [40] using the R package 'mediation' [41].

Given the heterogeneous nature of the medical data, a distinct survey methodology, specifically qualitative content analysis following the framework developed by Mayring, was used to examine the content of the medical documentation [42, 43]. This approach facilitated the comprehensive analysis of diverse documentation styles moreover allowing for the inclusion of latent content.

### Ethical considerations

Ethical principles concerning design and execution of the study were observed according to international standards. Most notably, the relevant principles of UNICEF document “Ethical Principles, Dilemmas and Risks in Collecting Data on Violence against Children” were applied. Pseudonymised retrospective data acquisition of court protocols and reports were viewed and recorded in the court by the study team. The assessment in the court premises took place in pairs of researchers. The two study members looked through, copied, and assigned together all relevant data. Furthermore, the approval of the responsible ethics committee was obtained.

## Results

### Progression of legal proceedings

During the time period July 2015 to June 2017, in total 233 children (mean age 7.14 years, 41.6% males) were referred to FOKUS and 209 (100%) children (age mean 7.79 years, 41.6% males) were potential victims for abuse. Of which 87 (41.6%) children (age mean 9.74 years, 33.3% males) were reported to the police.

**Fig. 1:**
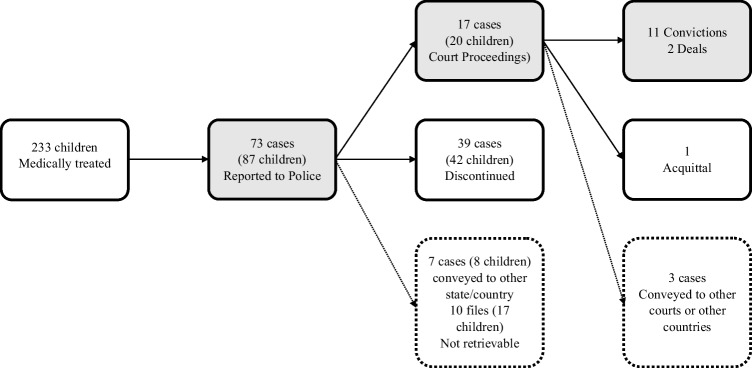
Follow-up of all cases

For the evaluation of the initiation of main proceedings, a total of 87 children’s cases were investigated. It was noticed that proceedings were terminated in Vienna if the jurisdiction of other provinces in Austria or other European countries were responsible (7 cases/ 8 children during preliminary proceedings and 3 cases during main proceedings). Moreover, in 17 children’s cases, the court files were not available (10 files/17 children). Of the remaining children’s cases, 20 children (32.3%) were subject to main proceedings (Fig. 1).

### Factors influencing reporting to the authorities by the medical professionals of FOKUS

Incidents were reported significantly more frequently to the police with female victims (*p* = 0.047), for sexual abuse (*p* = 0.012) and with victims older than 6 years (*p* = 0.017) (Table 1). Girls were significantly more often victims of sexual violence and boys more often victims of physical violence (*p* < 0.001) (Table 2). On the other hand, elder children were more frequently victims of sexual violence and younger children of physical violence (*p* < 0.001) (Table 3). Multiple logistic regression model related to the type of abuse, the gender, and age of the victims as independent variables could show that elder children reported significantly more frequently to the police (*p* < 0.001, Nagelkerke R^2^ = 0.158). Relation to the gender of the victims and type of the abuse that seemed to be significant in bivariate analyses were no longer significant (Table 4). Mediation effects were suspected and tested. In conclusion, age mediated the relationship between the gender of the victims and reporting to the police (*p* = 0.046) as well as the relationship between the type of abuse and reporting to the police (*p* < 0.001) (Table 5).

**Table 1:** Bivariate analyses in terms of police reporting

Parameter	Police report (*n* = 87)	No police report (*n* = 122)	Test statistic	*p*-value
Victim gender				
Boy	29 (33.3)	58 (47.5)	*F*(1, 172) = 3.996	0.047
Girl	58 (66.7)	64 (52.5)		
Type of abuse				
Physical abuse	30 (34.5)	68 (55.7)	*F*(1, 172) = 6.433	0.012
Sexual abuse	57 (65.5)	54 (44.3)		
Age	M = 6.39 SD = 4.81	M = 9.74 SD = 5.05	*F*(2.86, 489.52) = 5.550	0.001
[Newborn – 36 month]	8 (9.3)	33 (27)		
Between 3 and 6 years	17 (19.8)	33 (27)	*β* = 0.754 (0.480), *z* = 1.571	0.116
Between 6 and 12 years	28 (32.6)	36 (29.5)	*β* = 1.166 (0.489), *z* = 2.386	0.017
Between 12 and 18 years	33 (38.4)	20 (16.4)	*β* = 1.918 (0.514), *z* = 3.732	< 0.001
Missing	1			

Annotation. Numbers are absolute values (percentages), F-values, Pearson’s chi-square test with second-order correction of Rao and Scott (1992); β-values, regression slopes (SE) for age group versus baseline in squared brackets

**Table 2:** Analyses gender differences

Parameter	Boy (*n* = 87)	Girl (*n* = 122)	Test statistic	*p*-value
Age				
[Newborn – 36 months]	20 (23)	21 (17.4)	*F*(2.98, 510.24) = 1.417	0.237
Between 3 and 6 years	21 (24.1)	29 (24)		
Between 6 and 12 years	30 (34.5)	34 (28.1)		
Between 12 and 18 years	16 (18.4)	37 (30.6)		
Missing		1		
Type of abuse				
Physical abuse	57 (65.5)	41 (33.6)	*F*(1, 172) = 17.301	< 0.001
Sexual abuse	30 (34.5)	81 (66.4)		

Annotation. Numbers are absolute values (percentages); F-values, Pearson’s chi-square test with second-order correction of Rao and Scott (1992)

**Table 3:** Analysis of differences between type of abuse and age

Parameter	Sexual abuse (*n* = 111)	Physical abuse (*n* = 98)	Test statistic	*p*-value
Age			*F*(2.90, 499.68) = 7.952	< 0.001
[Newborn – 36 months]	7 (6.4)	34 (34.7)		
Between 3 and 6 years	30 (27.3)	20 (20.4)	*β* = 1.986 (0.515), *z* = 3.857	< 0.001
Between 6 and 12 years	37 (33.6)	27 (27.6)	*β* = 1.896 (0.512), *z* = 3.699	< 0.001
Between 12 and 18 years	36 (32.7)	17 (17.3)	*β* = 2.331 (0.535), *z* = 4.357	< 0.001
Missing	1			

Annotation. Numbers are absolute values (percentages); F-values, Pearson’s chi-square test with second-order correction of Rao and Scott (1992); β-values, regression slopes (SE) for age group versus baseline in squared brackets

**Table 4:** Multiple logistic regression model for police reporting

Parameter	β (SE)	z-value	*p*	Odds ratio
Intercept				
Type of abuse	0.433 (0.414)	1.045	0.296	1.542
Victim gender	0.331 (0.350)	0.948	0.361	1.392
Age	0.118 (0.035)	3.426	0.001	1.125

Annotation. β-values, regression slopes (SE)

**Table 5:** Mediation analyses of age in terms of police reporting

Mediation	Direct effect β (SE)	*p*	Additional mediation effect	*p*-value
Age mediation effect on victim gender x police report	0.096 (0.069)	0.162	0.041 (0.025)	0.046
Age mediation effect on type of abuse x police report	0.113 (0.075)	0.176	0.088 (0.031)	< 0.001

Annotation. β-values, regression slopes (SE)

### Influencing factors for the initiation of main proceedings

**Fig. 2:**
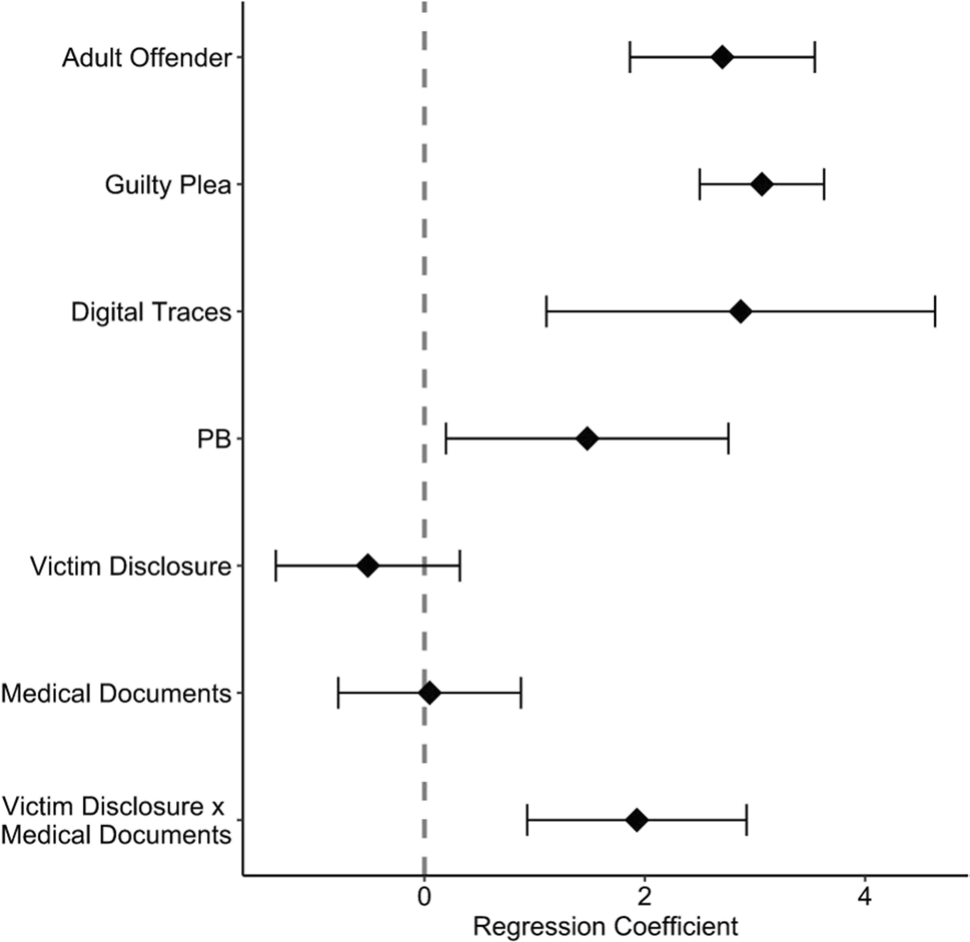
Interaction of medical documentation and victim disclosure on the probability of opening court proceedings

**Fig. 3:**
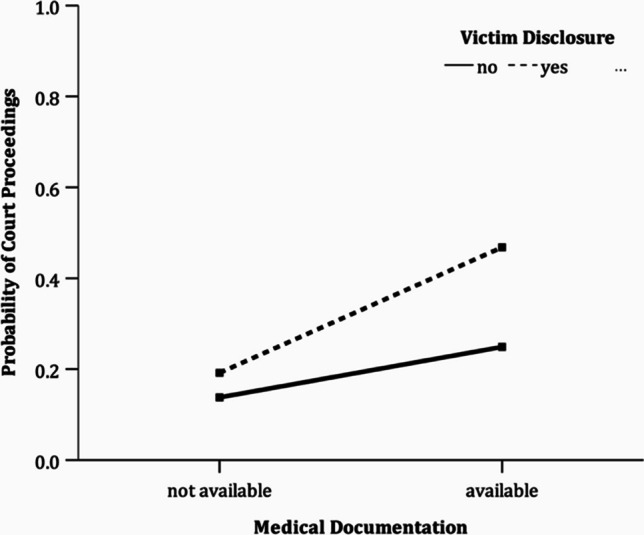
Coefficients of multiple logistic firth regression predicting opening court proceedings with 95% confidence intervals

Bivariate analyses showed that main proceedings were opened significantly more often if the offender was an adult (*p* = 0.001), admitted his guilt (*p* = 0.004), there were digital traces (*p* = 0.003) and there was a trial support (*p* = 0.013). There were no bivariate links between the opening of the main proceedings and the sex of the child, the age of the child, the sex of the offender, the relationship to the offender or the type of abuse. The presence of medical documentation was not significant and victim disclosure was hardly of any significant importance (*p* = 0.064) (Table 6). Multiple logistic regression analysis using significant independent variables and a theoretically based interaction of medical documentation and victim disclosure on initiation of main proceedings was performed. This showed a significant interaction of medical documentation and victim disclosure (*p* < 0.001) and was related to a higher probability of opening the main trial (Fig. 2). Admission of guilt (*p* < 0.001), digital evidence (*p* = 0.001), trial counseling (*p* = 0.024), and majority of the offender (*p* < 0.001) were also statistically significant for opening court proceedings in the multivariate model (Nagelkerke R^2^ = 0.698; Table 7 and Fig. 3).

**Table 6:** Bivariate analyses in terms of opening proceedings

Parameter	Prosecuted *n* = 20	Discontinued *n* = 42	Test statistic	*p*-value
Victim gender				
Boy	4 (20)	12 (28.6)	*F*(1, 55) = 0.587	0.447
Girl	16 (80)	30 (71.4)		
Gender perpetrator				
Male	12 (63.2)	29 (69)	*F*(1.89, 102.08) = 0.151	0.849
Female	2 (10.5)	5 (11.9)		
Both parents	5 (26.3)	8 (19)		
Missing	1			
Relationship victim perpetrator				
Foreign	4 (20)	2 (4.8)	*F*(1.99, 109.60) = 1.759	0.177
Closer relationship	6 (30)	20 (47.6)		
Related	10 (50)	20 (47.6)		
Type of abuse				
Physical abuse	6 (30)	14 (33.3)	*F*(1, 55) = 0.043	0.837
Sexual abuse	14 (70)	28 (66.7)		
Age			*β* = 0.08 (0.08), z = 0.987	0.324
Digital traces				
Yes	6 (30)	1 (2.4)	*F*(1, 55) = 9.790	0.003
No	14 (60)	41 (97.6)		
Victim disclosure				
Yes	18 (90)	29 (69)	*F*(1, 55) = 3.575	0.064
No	2 (10)	13 (31)		
Guilty plea				
Guilty	7 (35)	2 (4.8)	*F*(1, 55) = 9.175	0.004
Not guilty	13 (65)	40 (95.2)		
Trial support				
Yes	17 (85)	15 (35.7)	*F*(1, 55) = 12.068	0.001
No	3 (15)	27 (64.3)		
Adult offender				
Yes	20 (100)	30 (71.4)	*F*(1, 55) = 6.651	0.013
No	0	12 (28.6)		
Medical documentation				
Yes	15 (75)	23 (54.8)	*F*(1, 55) = 2.017	0.161
No	5 (25)	19 (45.2)		

Annotation. Numbers are absolute values (percentages); *F*-values, Pearson’s chi-square test with second-order correction of Rao and Scott (1992); *β*-values, regression slopes (*SE*) for age group

**Table 7:** Multiple logistic regression model for opening court proceedings

Parameter	β (SE)	z-value	*p*-value	Odds ratio
Intercept				
Adult offender	2.704 (0.428)	6.323	< 0.001	14.939
Guilty plea	3.063 (0.288)	10.650	< 0.001	21.392
Digital traces	2.871 (0.900)	3.191	0.001	17.655
Trial support	1.477 (0.654)	2.258	0.024	4.380
Victim disclosure	-0.514 (0.426)	-1.207	0.227	0.598
Medical documents	0.047 (0.423)	0.112	0.911	1.048
Victim disclosure x medical documents	1.928 (0.508)	3.796	< 0.001	6.876

Annotation. Firth-correction for logistic regression model; *β*, regression slopes

## Discussion

The aim of this study was to investigate and scientifically determine the factors influencing reporting and criminal proceedings in Austria in order to contribute to an increase in the currently low clearance rate in child abuse cases, which as a further consequence have the potential to lead to positive changes in the following areas:

- Safety of the victim and possible future victims by sentencing the perpetrator,
- Empowerment of the victim,
- Better reputation of the justice system, leading to trust and increased willingness to cooperate with the judicial bodies involved in the criminal proceedings [1–3].

International studies could show that the multi-step process leading up to a conviction involves the risk of early termination [4, 12]. The biggest hurdle in the judicial process, according to the findings of our study, proved to be for a case to be tried by the court. Only 23% of cases reported to the police were forwarded to the main proceeding supporting the findings of Cashmore et al. that only about one in five cases of child sexual abuse proceeds beyond the investigation stage [19].

Out of the 17 cases that were brought to trial, only one (6%) ended in an acquittal (3 cases were moved to other jurisdictions and therefore could not be investigated further).

This clearly shows that the point at which the conviction of the perpetrator ultimately fails is in most cases not the acquittal before the court, but takes place at an earlier stage: the vast majority of cases were already discontinued during the pre-trial proceedings. In this stage of the investigation many reported cases are dropped even if the initial suspicion and the (medical) evidence were provided. This also supports studies showing that even substantiated cases may not lead to prosecution [4]. In the context of forensic examinations by medical personnel, this indicates, for example, that when collecting evidence, the focus should not only be on court usability, but always keep in mind that the hurdle of reaching trial in the first place should be a priority.

The range of factors that influence the initiation of main proceedings in child abuse cases is diverse. In this study, a strong influence evidence types was observed indicating that certain evidence categories are more significant than others for the perpetrator to be prosecution before court.

### Admission of guilt

A guilty plea of the accused perpetrator has significant impact on prosecution, confirming previous findings [4, 19]. Prosecution occurs with a probability of 96% more likely with an admission of guilt than without.

### Digital evidence

Digital traces are becoming increasingly important in the prosecution and conviction of child abuse. (e.g., pictures and videos, as well as chat histories, sent by the perpetrator or victim documenting the abuse). Consequently, digital traces of abuse are a strong indicator for further investigations, persecution, and conviction. Nowadays, searching for digital evidence on smartphones and computers soon after a first suspicion arises should be standard procedure, especially as digital communication continues to permeate everyday lives of all age groups, including very young children. IT specialists working closely with the police can help secure this type of evidence. With digital tools becoming more and more prevalent, all involved bodies must keep up to date with current laws, as well as digital development (e.g. new chat and photo/video platforms) in order to provide the best possible evidence admissible in court. It is highly recommended that a concrete legal basis should be provided in order to safeguard both the rights of the accused, as well as work towards the best possible collection of relevant evidence. This is supported by previous findings [44, 45].

### Medical documentation

The role of medical documentation and evidence in the persecution process is discussable and depends on the obvious case related description. It is well known, that medical evidence of child abuse can rarely be linked to abuse with complete certainty [9–11, 17]. Theoretically, other explanations can be found for injuries and other physical signs of abuse [13, 16] This has also been the case in our study, as the analysis of medical documentation has proven:

o “Scars can either be self-inflicted or due to external factors”;
o “Infliction of third party under the above conditions cannot be excluded”;
o “Hymen: polyp at 7 o'clock on the edge of the hymen, old notch at 3 o'clock; no evidence or reason for sexual abuse.”
o “Rhagade anal at 1 o'clock, old and healed. According to Adams' classification for prepubertal girls, these are nonspecific signs of sexual assault that can neither prove nor rule out sexual assault.”
o “Scratch marks (allegedly from a cat), healed scar, scratch on the edge of the buttocks, hematoma on the elbow […]. Abuse cannot be eliminated.”
o “No tangible evidence of external force to the spine, blunt force (blows) cannot be ruled out from a forensic point of view.”

Physicians have learned to combine medical history and physical examination to define differential diagnosis. In cases of child abuse, medical documentation needs to follow forensic standards and to provide a clear description for the executive and judicial bodies. This means experts for child abuse and interdisciplinary teams are necessary to use standardized medical documentation and other forensic evidence. Besides possible medical uncertainty, reasons for not reporting include the lack of feedback on the part of the executive and judicial body or the possible reservations on having to testify the legal proceedings [46, 47]. In addition to the challenges posed by the absence of conclusive findings from medical examinations, there are instances where obtaining medical documentation is either not feasible or no longer possible. Various factors may contribute to hindrances in obtaining a usable medical documentation, such as victims refusing to undergo examinations, cases of abuse lacking visible or persistent physical manifestations, or incidents that transpired in the remote past, making it impractical to examine their consequences.

### Victim disclosure

Medical documentation with incomplete prove for child abuse can be supported by the victim’s disclosure, showing that combining several types of evidence can strengthen the case against the abuser. This finding is in accordance with earlier published studies, suggesting that children’s statements play a key role in proving child abuse because of the difficult examination process [11, 48, 49].

### Trial support

Another aspect that proved to have an influence on the continuation of legal proceedings is whether process guidance by an independent team of specially trained psychologists and psychotherapists accompanying the victims through the court process and reducing mental health implications [2, 50] was available.

This can be especially important, as child disclosure — proven to be an important source of evidence — is more likely successfully used as evidence if professional trial support is provided. The quality of the disclosure also improves if easily understandable and for children adapted guidelines for interviewing the victim are provided [51].

In conclusion, our results have demonstrated that admission of guilt, digital evidence, trial counseling, and majority of the offender are associated with a higher probability for opening court proceedings. Our findings furthermore support that those cases that reach the main proceeding — the final stage of the legal process before the conviction — are usually very well founded. Out of the 17 cases that were brought to trial, only one (6%) ended in an acquittal.

### Strength and limitations of the study

One of the strengths of this study is that the cases could be tracked from the initial examination by the medical professionals in the hospital to the conviction or termination of the proceedings. The aforementioned studies often concern either the phase until reporting or the phase starting from the police report.

A joint evaluation of cases of child sexual abuse and other forms of child abuse may facilitate a better understanding of the medical and legal process as a whole. Furthermore, the strong interdisciplinary approach both in the researcher team, as well as in the combination of medical and legal findings, provides an insight that many previous studies were not able to facilitate. Another strength is that children examined by FOKUS were provided with a quick and thorough medical examination by specifically trained medical professionals of various fields. The documents provided by FOKUS’ team members were specifically designed to be used in legal proceedings and to provide a comprehensive overview of the child’s injuries and possible course of events.

Limitations were that the information about cases which were not reported to the police was limited, as the team’s checklist was solely developed for the court files. The medical history of the other cases was not made available. This makes the study highly informative concerning the procedure of executive (police) and judicial bodies (district attorneys, judges), but does not give an in-depth insight in medical professionals’ decisions regarding whether to report or not. Also, data concerning perpetrators was naturally lacking in those cases in which the team was not provided with court files. Consequentially, the study only provides detailed analysis of those cases for which there are existing case files.

In Austria, the case files in criminal proceedings are currently in paper format, lacking standardization in their structure. Even though it is mandatory to incorporate specific elements in each case file, the medical documentation — if included — can significantly vary depending on the examining physicians. Despite exclusively focusing on cases assigned to FOKUS, which entail standardized documentation, the presence of medical records in the case file is not always consistent. Their contents, such as the types of medical examinations or the degree of clarity, are conditional on the specifics of each case. Due to these circumstances, determining which medical examinations may contribute to a higher conviction rate is not currently feasible.

Another limiting aspect was that a number of cases (as seen in the results section of the article) were moved to another jurisdiction in the course of the investigation, as new information concerning the deciding factors for jurisdiction were found.

Another obvious limitation of any study dealing with child abuse is that the dark figure of cases is assumed to be much higher than the number of cases analyzed.

## Conclusion

Our findings underline how challenging the successful persecution of an offender in cases of child abuse is. The most critical stage of the legal proceeding is not reaching a conviction within the main proceeding itself, rather for the case to progress to the final stage of proceedings in the first place. Only one case brought to trial ended in an acquittal (6%).

This clearly demonstrates that the drop-out rate within legal proceedings is not acquittal before court, but occurs at earlier stages, as the large majority of cases were terminated in the preliminary stage of the criminal proceeding and never reached a court hearing.

The results indicate that certain types of evidence have an increased influence on the initiation of legal proceedings, namely cases with admission of guilt or digital evidence were much more likely to end in a conviction than cases with other types of evidence. Besides evidence types, other factors such as psychological support of the victim through professional trial support may influence chances of a successful legal procedure.

### Supplementary Information

Below is the link to the electronic supplementary material.

Supplementary file1 (PDF 535 KB)

## Data Availability

The data are not publicly available for ethical and privacy reasons.

## Abbreviation

FOKUS: Forensic examination center for children and adolescents

## Footnotes

1 This is this is regulated in paragraph 37 of the Austrian Code of Criminal Procedure (§ 37 StPO).

2 For the few cases where evidence of neglect was encountered, the primary reason for referral to FOKUS was physical or sexual abuse.
