# Supplementary material for: Influence of various factors on the legal outcome of cases of child abuse—experiences gathered at an interdisciplinary forensic examination center in Vienna, Austria
Source: Int J Legal Med. 2023 Oct 12;138(1):3–14. doi: 10.1007/s00414-023-03094-y (PMC10772007; doi:10.1007/s00414-023-03094-y)
Supplement: Supplementary file 1 — Supplementary file1 (PDF 535 KB) [file 414_2023_3094_MOESM1_ESM.pdf]

Erhebungsbogen bei Verdacht auf Kindesmisshandlung

Name des Kindes: ______________________________

Geburtsdatum: ______________________________

Geschlecht: ☐ männlich ☐ weiblich ☐ divers

Adresse: ______________________________

____

Patientenetikett

Staatsbürgerschaft: ______________________________ SV-Nummer: ______________________________

Sprachliche Verständigung:
Sprache: ______________________________
☐ fließend ☐ gebrochen ☐ Dolmetsch ________________(Name) ☐ nicht möglich

Derzeitiger Aufenthaltsort des Kindes: ______________________________

Obsorgeberechtigte/r bei Erstvorstellung: ______________________________

Zuständige Stelle der Kinder- und Jugendhilfe (falls bekannt): ______________________________

☐ ambulant ☐ stationär Aufnahmezahl: ______________________________

Verdacht auf: **Verdacht richtet sich gegen:** ______________________

☐ Vernachlässigung ☐ Körperliche Misshandlung ☐ Münchhausen Syndrom by proxy

☐ Sexueller Missbrauch ☐ Psychische Misshandlung

Patient*in wird gebracht durch (Name, Adresse, Telefonnummer, Bezug zum Kind): ______________________________

Bei der Untersuchung externe anwesende Personen: ______________________________

weitere anwesende Personen: ______________________________

Primäre Verdachtsäußerung / Meldung durch (Name, Adresse, Telefon, Bezug zum Kind): ______________________________

⚠ Zu erfragen sind: **Wer? Was? Wie? Wo? Wann? Weshalb?** In Form von **offenen Fragen**; Ja/ nein Fragen vermeiden, **keine Suggestivbefragung!**

Sachverhaltsdarstellung (Grund für den Arztbesuch, Unfallhergang/Verletzungsursache, Zeitpunkt): ☐ siehe ergänzende Dokumentation

Auffällige Befunde/Verletzungen (→ Siehe Körperschema):

Auffällige Beobachtungen (Verhalten, Kontakt zur Bezugsperson):

Erklärung des Kindes (in genauem Wortlaut):

Erklärung der Eltern, Erziehungsberechtigten und/oder Begleitpersonen (in genauem Wortlaut):

Angaben zu weiteren Kindern im gemeinsamen Haushalt (Anzahl, Alter, Geschlecht, Einschätzung der Gefährdung):

Bisherige Untersuchungen (z.B. Labor, Bildgebung, Konsil etc. als Beilagen):

Laufende bzw. durchgeführte Maßnahmen: ☐ keine Maßnahmen ☐ Gefahr in Verzug ☐ Sonstiges: ______________________________

Sicherheit des Kindes: ☐ gewährleistet ☐ nicht gewährleistet

Polizeiliche Anzeige: ☐ erfolgt ☐ nicht erfolgt (Begründung angeben) ______________________________

↳ Wer hat Anzeige erstattet: __________________________________ ↳ Wann wurde Anzeige erstattet: : ______________________________________

Gefährdungsmeldung an die Kinder- und Jugendhilfe: ☐ Ja ☐ Nein

↳ Wer hat Meldung erstattet: __________________________________ ↳ Wann wurde Meldung erstattet: ______________________________________

↳ Ausfolgeverbot: ☐ Ja (Datum, Uhrzeit) _____________ ☐ Nein

Obsorgeberechtige/r informiert: bezgl. der Meldung an: ☐ Polizei ☐ Kinder- und Jugendhilfe ☐ Kinderschutzgruppe

Merkmale der Vernachlässigung: (z.B. Zahnstatus, Haut, Kleidung, Körperhygiene, Ernährungszustand, psychische Auffälligkeiten, Interaktion, Verhalten):

Fotodokumentation (immer in Verbindung mit schriftlicher Befunddokumentation und Eintragung in Körperschema!)

Wurde eine Fotodokumentation durchgeführt? ☐ ja ☐ nein ggf. Anzahl der Aufnahmen:

wenn „nein", warum nicht: __________________________________ wenn „ja", wo archiviert: ____

⚠ Bei frischen Verletzungen ist eine Fotodokumentation im Verlauf über Tage anzustreben! Immer Bildserien mit zunehmend detailreicherem Bildausschnitt anfertigen (Übersichts-, Detail- und Nahaufnahmen). Ein manueller Weißabgleich zu Beginn gewährleistet die korrekte Farbwiedergabe. Übersichtsaufnahme mit einwandfrei identifizierbarem Gesicht des Patienten anfertigen. Nahaufnahmen mit und ohne Maßstab (immer in Abbildungsebene!) ggf. mit der Makrofunktion der Kamera erstellen. Beschädigung und Verunreinigung der Kleidung möglichst vor dem Entkleiden fotografieren. Der topografische Bezug der abgebildeten Verletzungen und Spuren muss jederzeit erkennbar sein (ggf. aus mehreren Blickwinkeln fotografieren). Verletzungen immer in ungereinigtem und in gereinigtem Zustand fotografieren.

Wichtig: Der Fotodokumentation der Befunde ist genügend Zeit einzuräumen, da ihr bei späteren Begutachtungen eine große Bedeutung zukommt!

Spurensicherung/Asservate

Beschädigungen an der Kleidung (Foto!)? ☐ nein ☐ ja:

Verunreinigungen der Kleidung (Blut, Sekret etc.)? ☐ nein ☐ ja:

Kleidung sichergestellt (→ einzeln in **Papiersäcke** verpacken u. Beschriften!) ☐ nein ☐ ja:

Andere Spuren/Fremdmaterial vorhanden? ☐ nein ☐ ja, folgende:

☐ Blutverdächtige Anhaftungen (Abrieb) ☐ Fasern/Haare (Papiersäckchen)

☐ Sekretspuren (Abrieb) ☐ Schmutz/botanisches Material (Abrieb od. Papiersäckchen)

☐ Speichelspuren (Abrieb) ☐ Fremdkörper (Papiersäckchen)

☐ Fingernagelschmutz (Abrieb) ☐ Verklebte Haare (abschneiden u. in Papiersäckchen sichern)

☐ Sonstige: ____

Vergleichsproben (Patient*in):

☐ Blut ☐ Mundhöhlenabstrich (Abrieb)

Weitergabe der Beweismittel / Asservate (Abstriche, Kleidung, Tatmittel etc.):

Verletzungsdokumentation/Spurensicherung (Art, Beschreibung, Lokalisation bitte einzeichnen):

Hinweise:

Säuglinge u. Kleinkinder (0-6 Jahre):
Hämatome, Ekchymosen, Schwellungen, nässende Wunden und Schürfungen an Kopf und behaarter Kopfhaut, Gesicht, Hals, Schütteltrauma, Koma, Krämpfe, Griffmarken – Oberarme und seitlicher Thorax, unklare paroxysmale Episoden

Schulkinder (6-14 Jahre):
entsprechende Befunde an Gesäß, Rücken, Beinen sowie Damm, Genitalien, After, Oberschenkel und Unterbauch (letzeres bes. bei sexuellem Missbrauch)

Jugendliche (14-18 Jahre):
Kopf, Hals, sowie insbesondere an bedeckten Körperbereichen wie Schultern, Nacken und Oberarmen. Genital/Analbereich

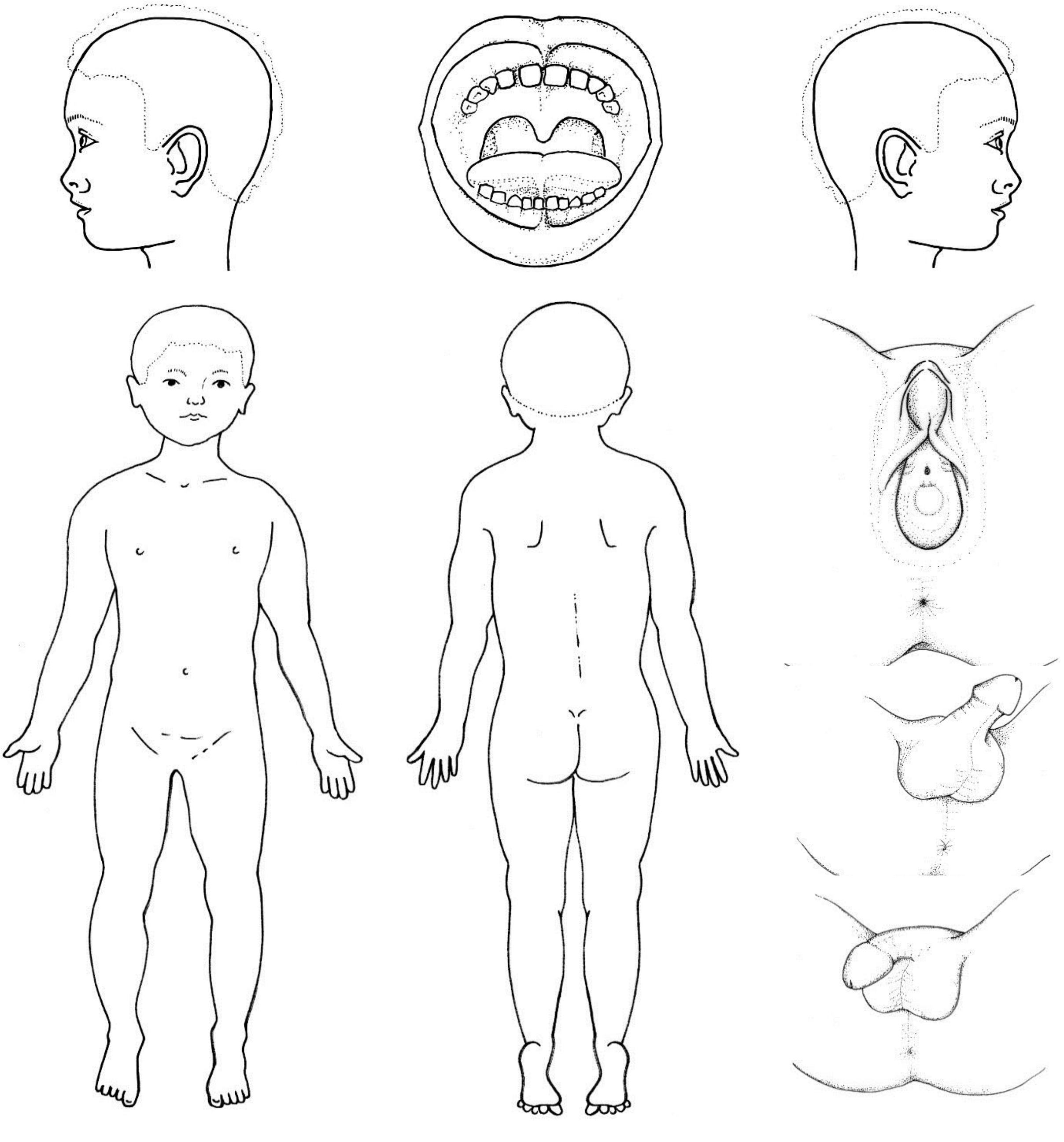

Wichtig ist eine systematische **Ganzkörperuntersuchung** (inkl. behaarter Kopfhaut, Retroaurikulärregion, Mundhöhle, Augenbindehäute, Innenseite der Arme und Schenkel). Auf Befunde wie Hämatome, Schürfungen, Petechien, Strangulationszeichen, Biss- u. Griffmarken sowie geformte Verletzungen achten! **Frühzeitige Fotodokumentation** anstreben (Übersichts-, Näherungs- u. Detailaufnahmen mit Maßstab)!
Alter der Verletzung nach Farbe miteinbeziehen: Tag 1 rot/blau; Tag 2 blau/purpur; Tag 6 grün; Tag 8 gelb/braun; Tag 10 beseitigt;
wichtig*:* Farbe variiert je nach Tiefe, Größe und Lokalisation der Verletzung. **Verschiedenfarbige Hämatome** sind verdächtig!

Bemerkungen:

Weitere Maßnahmen (empfohlen):

Datum, Ort

Name und Unterschrift der untersuchenden Fachkraft
